# An extended model of vesicle fusion at the plasma membrane to estimate protein lateral diffusion from TIRF microscopy images

**DOI:** 10.1186/s12859-017-1765-y

**Published:** 2017-07-24

**Authors:** Antoine Basset, Patrick Bouthemy, Jérôme Boulanger, François Waharte, Jean Salamero, Charles Kervrann

**Affiliations:** 1 Inria, Campus de Beaulieu, Rennes, 35042 France; 2 Institut Curie, PSL Research University, CNRS UMR 144 and PICT-Cell and Tissue Imaging Facility, 12 rue Lhomond, Paris, 75005 France; 3 CNES, 18 avenue Edouard Belin, Toulouse, 31401 France; 4 MRC Laboratory of Molecular Biology, University of Cambridge, Francis Crick Avenue, CBC Cambridge Biomedical Campus, Cambridge, CB2 0QH UK

**Keywords:** TIRF microscopy, Vesicle fusion model, Molecule diffusion, Protein release rate, Model fitting, Exocytosis, Transferrin receptor (TfR), Langerin protein

## Abstract

**Background:**

Characterizing membrane dynamics is a key issue to understand cell exchanges with the extra-cellular medium. Total internal reflection fluorescence microscopy (TIRFM) is well suited to focus on the late steps of exocytosis at the plasma membrane. However, it is still a challenging task to quantify (lateral) diffusion and estimate local dynamics of proteins.

**Results:**

A new model was introduced to represent the behavior of cargo transmembrane proteins during the vesicle fusion to the plasma membrane at the end of the exocytosis process. Two biophysical parameters, the diffusion coefficient and the release rate parameter, are automatically estimated from TIRFM image sequences, to account for both the lateral diffusion of molecules at the membrane and the continuous release of the proteins from the vesicle to the plasma membrane. Quantitative evaluation on 300 realistic computer-generated image sequences demonstrated the efficiency and accuracy of the method. The application of our method on 16 real TIRFM image sequences additionally revealed differences in the dynamic behavior of Transferrin Receptor (TfR) and Langerin proteins.

**Conclusion:**

An automated method has been designed to simultaneously estimate the diffusion coefficient and the release rate for each individual vesicle fusion event at the plasma membrane in TIRFM image sequences. It can be exploited for further deciphering cell membrane dynamics.

## Background

Characterizing dynamic protein behaviors in live cell fluorescence microscopy is of paramount importance to understand cell mechanisms. In the case of membrane traffic, cargo molecules are transferred from a donor to an acceptor compartment [1]. For instance, during the exocytosis process, a vesicle conveys cargo molecules to the plasma membrane, and then opens to expel them from the cell. Total internal reflection fluorescence microscopy (TIRFM) is particularly well suited for focusing on the late steps of exocytosis events, which occur at the plasma membrane [2]. However, it is still a challenging task to quantify local dynamics of proteins, and in particular, to estimate the local behavior of the transmembrane proteins which are also transported through the exocytic vesicles, once the fusion event had occurred at the plasma membrane. The type of dynamics undergone by transmembrane proteins in the plasma membrane is usually assumed to be a lateral free diffusion [3], at least within a short time scale, which is the case for the cell mechanisms we are interested in. Physical barriers depending on the nature of interactions of these proteins with their local environment, which impede the free diffusion of molecules in the plane of the membrane, may impose diverse levels of segregation [4].

In this paper, we investigate protein dynamics issues attached to exocytosis events observed in TIRF microscopy. More precisely, we focus on the dynamics of two fluorescently labeled cargo proteins, namely the Transferrin receptor (TfR), and a C-type Lectin, the Langerin. TfR and Langerin transmembrane proteins are inserted in cellular membranes, including the plasma membrane, and are involved in several biological processes. They are constitutively endocytosed and recycled through partly common endosomal-recycling pathways [5]. In the exocytosis-recycling step they are transported by a recycling carrier, which fuses to the plasma membrane. Then, the transmembrane proteins eventually diffuse in the augmented two dimensional lipid bilayer but may also remain temporarily bounded, forming semi-persistent structures slowly fading over time as a result of a dissociation process. In what follows, we discuss related work on diffusion quantification, simulation, and modeling, while positioning our approach with respect to the literature.

### Diffusion quantification

Regarding diffusion quantification, many methods were proposed to compute the diffusion coefficient. They can be classified into four main categories.

- Methods based on single particle tracking (SPT), that is, exploiting trajectories or successive displacements [6–9]. The diffusion coefficient is inferred from the mean squared displacement (MSD) assuming Brownian motion. An alternating criterion, maximum *a posteriori* (MAP), is used in [10].
- Fluorescence fluctuation spectroscopy, which relies on the spatial and/or temporal intensity correlation between spatially and/or temporally neighboring pixels [11–13].
- Maximum likelihood estimation based on the diffusion equation [14, 15]. The maximum likelihood estimation adopted in [15], assumes multiplicative log-normal measurement noise. Yet, results were provided only on simulated data, the reported work focusing mainly on model parameter identifiability.
- Intensity fitting methods in which an intensity model is formulated and estimated in a space-time volume of the microscopy image sequence [16–18], as exploited in fluorescence recovery after photobleaching (FRAP) experiments [18].

### Diffusion simulation

Simulations of lateral diffusion processes were exploited in [19] to improve the accuracy in evaluating FRAP measurements for the estimation of diffusion coefficients. In [20], simulations of both isotropic and anisotropic diffusion were defined on curved biological surfaces, and applied to the membrane of endoplasmic reticulum. A numerical method is also designed in [21] for computing diffusive transport on complex surface geometries from image data. Interactions between proteins and membrane structures were taken into account in [22, 23]. In contrast, since our method is able to locally estimate the parameters of interest by taking into account only a small space-time area around the vesicle fusion location, local homogeneity and planarity of the membrane can be reasonably assumed.

### Vesicle fusion modeling

**Fig. 1:**
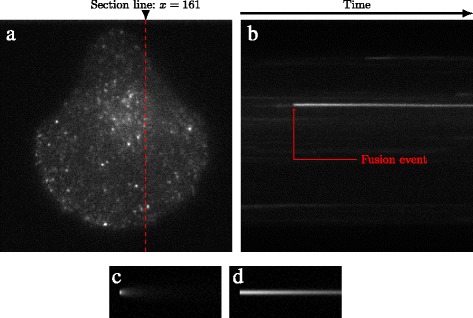
Comparison of a real vesicle fusion event in a TIRFM image sequence with simulations of the point source and SSED models. **a** First frame of a TIRFM image sequence. **b** Kymograph at column *x*=161 where one fusion event takes place (M10 cell expressing Langerin-pHluorin). **c** Kymograph obtained for a simulation based on the point source model (with *D* = 0.5 px^2^/f). **d** Kymograph obtained for a simulation based on the proposed SSED model (with *κ* ^−1^=100*f* and *D*=0.5px^2^/f)

Efforts have been undertaken to model diffusion in the plasma membrane after vesicle fusion. It was addressed in [16, 17]. In these works, the simple point source model was adopted, meaning that all the proteins are assumed to be initially concentrated in one single point and immediately diffused. This model thus relies on restrictive hypotheses which may yield non-accurate results. This is illustrated in Fig. 1 with kymographs. A kymograph gives the evolution over time of a given image column (or line), by concatenating its successive profiles. The horizontal axis represents time. Figure 1a contains the first frame of a TIRFM image sequence and the kymograph corresponding to column 161 where a Langerin fusion event takes place. The kymograph obtained for a simulation based on the point source model (Fig. 1b left) significantly departs from the real one. In contrast, the extended model we propose correctly mimics the real one (Fig. 1b right).

### Our approach

We propose an original vesicle fusion model, relying on two realistic hypotheses. First, we only assume that the vesicle is smaller than the radius *σ*_PSF_ of the microscope point spread function (PSF). Secondly, we take into account that the proteins are progressively released in the plasma membrane after the fusion occurs. As explained later, we model the release process as an exponential decay of the number of proteins contained in the vesicle. Hence, we term “small-extent source with exponential decay release” (SSED) the proposed model. Besides, our model fitting method is local both in space and time, allowing for the estimation of local protein dynamics for each individual vesicle fusion event. Both translational and rotational diffusion were handled in [24, 25]. However, the rotational component was shown to be negligible with respect to the lateral component [26]. As a consequence, we will address lateral diffusion only.

In the 2D TIRFM images we deal with, individual proteins cannot be resolved since they are too close from each other compared to the microscope resolution, which precludes SPT methods. Also, fluorescence fluctuation spectroscopy methods, such as Spatio-Temporal Image Correlation Spectroscopy (STICS) [27], assume spatial and/or temporal stationarity to a sufficient extent, and imply that all the proteins undergo a Brownian motion. In contrast, both spatial and temporal stationarity hypotheses are no more required for our method, since the estimation of the diffusion coefficient remains local in space (within a small patch) and time (over a few frames). Furthermore, our SSED model can accomodate mixed behaviour, that is, a portion of proteins remaining static for a while. Finally, among intensity fitting estimation methods, [17] showed leading performance for the point source model, but it is no more adapted to estimate the SSED model parameters. Therefore, we have defined a more elaborate method.

## Methods

### Point source fusion model

Before introducing our SSED model, let us first consider the point source fusion model. The mathematical model *u*(*p*,*t*) of the image intensity at point *p*∈*Ω* and time *t*∈[0,*T*] is fully determined by three items: *i)* the source particle distribution, *ii)* the evolution model, *iii)* the observation model. The source distribution defines both the spatial distribution of the particles before they start diffusing, and the law governing their release time to the plasma membrane or cytosol. The particle evolution model is the mathematical description of the motion of the proteins after fusion. Here, it is assumed to be Brownian, and consequently, lateral diffusion is the dynamical model governing the evolution of the whole particle population. The observation model is subdivided into several components, including possibly different noises and the optical transfer function or microscope PSF. We will first consider a noise-free observation model to specify the intensity model.

To move from Brownian motion to lateral diffusion, the concept of local concentration must be introduced. In the vesicle, and later in the cytosol or plasma membrane, particles are numerous, so that in TIRFM images we do not locally observe a single particle, but a population of *n* particles. Concentration is generally defined as the number of particles in a given local area.

The total concentration is the sum of the *source* concentration *C*_*s*_ and the *diffusive* concentration *C*_*d*_:

1 $$  C(p,t) = C_{s}(p,t) + C_{d}(p,t).  $$

The point source model assumes that all particles are initially concentrated at *p*_0_ and all instantaneously diffuse at time *t*_0_. Then, we can write:

2 $$  C(p,t) = \left\{ \begin{array}{ll} C_{s}(p,t) & \text{for} \; t=t_{0} \\ C_{d}(p,t) & \text{for} \; t>t_{0} \end{array} \right.  $$

and the source concentration distribution is proportional to a spatiotemporal Dirac distribution:

3 $$  C_{s}(p,t) = C_{0} \, \delta(p-p_{0}) \delta(t-t_{0}).  $$

The Fick’s second law [28] specifies the evolution over time and space of the local concentration as a function of the diffusion coefficient *D*:

4 $$  \frac{\partial C_{d}}{\partial t}(p,t) = D \Delta C_{d}(p,t),  $$

where *Δ* denotes the Laplacian operator. The Fick’s second law can be solved by Fourier analysis, which yields the following closed form Green’s function *Φ* defined on the domain *Ω*:

5 $$ \begin{aligned} \Phi(p,t) = \frac{1}{4\pi D(t-t_{0})} \exp\left({-\frac{\left\|{p-p_{0}}\right\|^{2}_{2}}{4D(t-t_{0})}}\right), \\ \forall t > t_{0}, p\in \Omega. \end{aligned}  $$

By linearity of the Fick’s second law, the concentration *C* is merely obtained by multiplying *Φ* by *C*_0_. Equation (5) can also be interpreted from a stochastic perspective as reflecting the probability of finding particles at position *p* and time *t*, if they undergo a Brownian motion of diffusion coefficient *D* and are initially concentrated at *p*_0_.

Let us now handle the observation model. Parameters of the intensity model are *C*_0_, the initial concentration at *p*_0_, the diffusion coefficient *D* and the radius *σ*_PSF_ of the PSF. To infer the intensity model *u*(*p*,*t*), we need to incorporate the observation model, reduced to the PSF and gain of the microscope. Since we are concerned with 2D membrane diffusion, the PSF can be restricted to a two-dimensional Gaussian function [29] of variance $\sigma _{\text {PSF}}^{2}$. The intensity model *u* is thus obtained by convolving the concentration *C* with a Gaussian kernel of variance $\sigma _{\text {PSF}}^{2}$:

6 $$\begin{array}{*{20}l}  u(p,t) \propto & \frac{C_{0}}{4\pi D(t-t_{0}) + 2\pi \sigma^{2}_{\text{PSF}}}  \\ & \exp\left(-\frac{\left\|p-p_{0}\right\|^{2}_{2}}{4D(t-t_{0}) + 2\sigma^{2}_{\text{PSF}}}\right). \end{array} $$

For the sake of simplicity, we introduce the constant *A*_0_ such that:

7 $$\begin{array}{*{20}l}  u(p,t) = & \frac{A_{0}}{2D(t-t_{0}) + \sigma^{2}_{\text{PSF}}}  \\ & \exp\left(-\frac{\left\|p-p_{0}\right\|^{2}_{2}}{4D(t-t_{0}) + 2\sigma^{2}_{\text{PSF}}}\right). \end{array} $$

### SSED fusion model

Our new SSED model introduces a continuous release of the proteins, meaning that each protein is expected to stay at the fusion location *p*_0_ during a certain amount of time after *t*_0_. This is expressed by an exponential decay of the source protein concentration inside the vesicle.

*C*(*p*,*t*) still represents the local protein concentration at point *p*∈*Ω*, where *Ω* is the image domain, and at time *t*, *t*∈[0,*T*]. As specified in Eq. (1), it is the sum of the source concentration component *C*_*s*_ and the diffusing concentration component *C*_*d*_. Now, the continuous release introduces a flow between the source concentration component *C*_*s*_, and the diffusing concentration component *C*_*d*_. The usual Fick’s second law is accordingly modified as follows:

8 $$  \frac{\partial C_{d}}{\partial t} (p,t) = D\,\Delta{C_{d}}(p,t) - \frac{\partial C_{s}}{\partial t}(p,t),  $$

subject to

9 $$\begin{array}{*{20}l}  C_{s}(p,t) &= C_{0} \, \delta (p-p_{0}) \exp(-\kappa(t-t_{0})) \,, \end{array} $$

where *κ* denotes the release rate, *δ*(*p*−*p*_0_)=1 if *p*=*p*_0_ or 0 otherwise, and *C*_0_ is the initial concentration at time *t*_0_. The exponential decay release is typically used in the representation of molecule dynamics in different configurations such as a narrow escape [23, 30] or a dissociation-like process [31, 32].

Let us still denote by *u*(*p*,*t*) the true intensity yielded by the SSED model at *p* in the *t*-th image. Using the superposition principle, and combining (8) and (9), we come up with the expression of *u* corresponding to the SSED model. More precisely, the Fick’s second law (8) can be solved by Fourier analysis, yielding closed-form Green’s function. Then, convolving the Green function with the microscope PSF and the source concentration (9), we get the following expression:

10 $$\begin{array}{*{20}l}  {}u(p,t) & = \frac{A_{0}}{\sigma^{2}_{\text{PSF}}} \exp\left(-\kappa t-\frac{\left\|p-p_{0}\right\|^{2}_{2}}{2\sigma^{2}_{\text{PSF}}}\right) \\ & \quad+ \int_{t_{0}}^{t} \frac{\kappa A_{0}}{2D (t-u) + \sigma^{2}_{\text{PSF}}} \\ &\quad\exp\left(-\kappa(u-t_{0})-\frac{\left\|p-p_{0}\right\|^{2}_{2}}{4D (t-u) + 2\sigma^{2}_{\text{PSF}}}\right) \mathrm{d}{u} \end{array} $$

where the factor *A*_0_ is related to the microscope PSF and the initial number of proteins in the vesicle. The integral in (10) is numerically evaluated, using a trapezoid integration with an adaptive step size.

Regarding the small-extent source configuration corresponding to the spatial vesicle area, we mathematically demonstrated that (10) is still valid for a non-pointwise source, if the radius of the vesicle is small enough with respect to *σ*_PSF_.

### Detection of fusion events

**Fig. 2:**
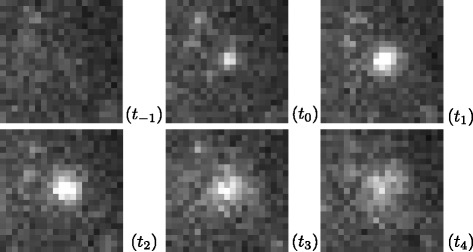
Sequence of patches cropped from a real TIRFM image sequence showing the appearance of the vesicle spot and its progressive temporal evolution during vesicle fusion to the membrane

To motivate our fusion event algorithm, we show a typical real example in Fig. 2. Figure 2 contains a sequence of image patches cropped at the same location and at distant time points from a real TIRFM image sequence. A bright spot suddenly appears at time *t*_0_ when the vesicle begins to fuse to the membrane. Then, the vesicle is gradually diffusing in this example.

Before estimating release rate *κ* and diffusion coefficient *D*, we need to detect the fusion events in the TIRFM image sequence, i.e., the event in which the transmembrane protein of interest is released to the plasma membrane.

Let us denote by *t*_0_ the time step when the event appears at point *p*_0_ in the image domain *Ω*. In this study, the transmembrane protein Transferrin receptor (TfR) is fluorescently labeled with a pH-sensitive probe, the pHluo-rin. Before *t*_0_, pH inside the vesicle is acidic, leading to very low pHluorin photon emission. When the vesicle fuses to the plasma membrane, the pHluorin gets exposed to the neutral extracellular medium, so that the fluorescence suddenly increases. As a consequence, we have to detect a localized rapid increase of intensity in the image *f*(*t*). We rely on the temporal backward difference *χ*_*f*_ defined as:

11 $$  \forall p \in \Omega, t > 0, \;\; \chi_{f}(p, t) = f(p, t) - f(p, t-1).  $$

A fusion event is perceived as a bright spot centered at point *p*_0_ in the map *χ*_*f*_(*t*_0_). We apply to every temporal difference map *χ*_*f*_(*t*) the spot detection method ATLAS [33]. It is based on the Laplacian of Gaussian (LoG) operator and proceeds in two steps. First, the scale *s* of the vesicles is automatically selected in a multiscale representation of the images. To determine it, we use the first ten frames of the input image sequence *f*, as it contains more spots than one frame of the *χ*_*f*_ sequence. Secondly, appearing spots related to a fusion event are detected by thresholding the LoG, at scale *s*, of *χ*_*f*_(*t*). The threshold automatically adapts to local LoG statistics estimated in a sliding Gaussian window, whose size is not critical. The detection threshold is inferred pointwise from a probability of false alarm fixed to 10^−6^. We come up with a set of *N* spots detected over the image sequence.

Regarding the TIRFM image sequences depicting Langerin, Langerin is tagged with the enhanced yellow fluorescent protein (EYFP). The EYFP is also a pH sensitive probe. It has the same type of behavior as pHluorin at the fusion time step. Consequently, we apply the same method to detect fusion events in Langerin image sequences, even if a less contrasted temporal intensity switch is observed. The difference in behavior occurs after the fusion time step in the release stage, as shown in Fig. 5.

Space-time location of the *i* ^*th*^ fusion event is denoted by *e*_*i*_=(*p*_0*i*_,*t*_0*i*_), where *p*_0*i*_, resp. *t*_0*i*_, is the location, resp. time instant, at which the *i*-th vesicle fusion occurs. Let *N* be the total number of detected fusion events in the TIRFM image sequence. Then, *N* spatiotemporal cuboids, $\{\mathcal {V}_{i},i=1,N\}$, are extracted around the *e*_*i*_’s, in which the background (structures and static spots) is estimated and removed [17]. We consider cuboids of 21×21 pixels in the spatial domain *Ω* and of 20 frames long (from *t*_0*i*_ to *t*_0*i*_+19) over the temporal axis. We come up with a set of *N* estimated foreground patch sequences $\widehat {\mathrm {z}_{i}},i=1\,..\,N$, in which only the central diffusing spot remains.

### Estimation of the biophysical parameters

Let us now focus on the estimation of the intensity model parameters in each reconstructed patch sequence $\widehat {\mathrm {z}_{i}}$. The intensity model corresponding to the SSED model, is defined by Eq. 10. It involves one more parameter (the release rate *κ*) than the point source model, and its expression is more complex. We were not able to satisfyingly estimate the SSED model parameters in simulated sequences using the estimation procedure we described in [17]. We need to design a more elaborate algorithm, described below.

For each detected fusion event *e*_*i*_, we have to fit the intensity model (10) derived from the SSED model, to the observed image intensities forming each patch sequence $\widehat {\mathrm {z}_{i}}$ reconstructed in subvolume $\mathcal {V}_{i}$. We assume that the observed intensity (after background subtraction) ${\widehat {z_{i}}}$, in the acquired microscope images, is given by the true intensity *u*, specified by the SSED model, corrupted by an additive zero-mean Gaussian noise. As a consequence, we can adopt the following quadratic function to estimate the model parameters:

12 $$  \mathcal{J}(p_{0},A_{0},\sigma_{\text{PSF}},\kappa,D) = \sum_{p\in\mathcal{V}_{i}} \left\| {\widehat{z_{i}}}(p,t) - u(p,t) \right\|^{2}.  $$

Model fitting will be achieved by minimizing $\mathcal {J}$ with respect to the model parameters *p*_0_,*A*_0_,*σ*_*PSF*_,*κ*,*D*. The minimization of function $\mathcal {J}$ has no closed-form solution, but it can be numerically solved in an iterative way. It turned out that the Gauss-Newton algorithm did not always converge to a satisfying minimum in our first experiments. Therefore, we have adopted the Levenberg-Marquardt algorithm along with the update scheme of [34]. Moreover, since the intensity model at *t*=*t*_0_ is a Gaussian spot, we can reliably estimate *p*_0_, *A*_0_ and *σ*_PSF_ by fitting a Gaussian spot model to the first patch (i.e., the one at *t*_0_) of the reconstructed sequence $\widehat {\mathrm {z}_{i}}$. Regarding *p*_0_, this step supplies a refinement of the value given by the fusion event detection algorithm. This way, the remaining two parameters *κ* and *D* can be estimated with a regression operating in two dimensions only.

In the estimation procedure, the initialization of the model parameters, and in particular the initialization of *κ*, is influential. Instead of estimating the parameters only once for each detected fusion event, we propose to start with different initializations of the parameters. After running the optimization algorithm, we select the run which minimizes the sum of squared residuals. In practice, as a tradeoff between accuracy and computation time, we have chosen the set {0.1,0.31,1,3.1,10} of initial values for *κ* ^(init)^ and {0.1,10} for *D* ^(init)^. In order to discard wrongly detected fusion events, or even badly fitted fusion models, we perform a chi-square goodness-of-fit test with a rate of type I error *α*=5*%*. Indeed, it was preferable to overdetect fusion events in order to ensure as few as possible missed fusion events, and then use this test to *a posteriori* remove false detections.

## Results and discussion

### Quantitative evaluation of the method performance

To evaluate the proposed estimation method, 300 synthetic patch sequences of size 21×21 pixels and length 20 frames were generated with different parameters to mimic real fusing spots $\widehat {\mathrm {z}_{i}}$’s. We have randomly set the diffusion coefficient in the range of 0.1 to 10 px^2^/f (px denotes the pixel pitch and f the frame period), and choose the PSF variance from 0.5 to 1.5 px^2^. As for the release rate *κ*, it varies between 0.1 and 10 f^−1^. The signal-to-noise ratio (SNR) ranges from 1 to 10.

**Fig. 3:**
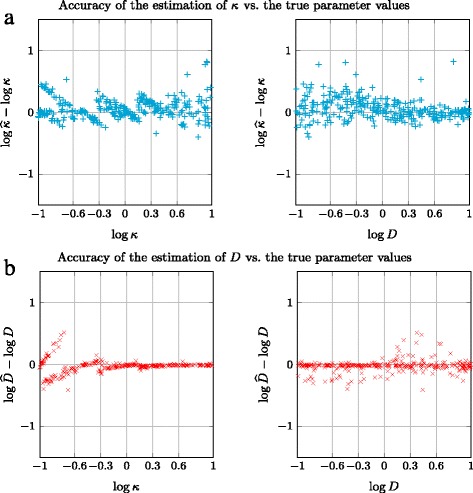
Accuracy of the estimation of *κ* (**a**) and *D* (**b**) on simulated sequences representing the SSED model

Logarithmic errors on the estimation of both *κ* and *D* are reported in Fig. 3 for each sequence. The estimation of *κ* is less accurate than that of *D*, but we will see in the next subsection that the accuracy is largely sufficient to extract relevant information from real TIRFM images. Moreover, large errors are very rare. Over the 300 generated sequences, only 5 have an absolute logarithmic error higher than 0.5, and the mean absolute logarithmic error (MALE) is quite low, it is equal to 0.12.

More or less periodic effects can be observed in the upper left plot of Fig. 3. They are due to clusters of suboptimal estimators corresponding to the same local minimum for a group of *κ* values, close to the values of this group of simulated *κ* values. Indeed, these “descending slanted alignments” could be approximated by a straight line of equation *y*=*a*−*x*, where *x* stands for log*κ* and *a* is a constant. This undesirable effect is mainly related to the initialization issue. By the way, our experiments on artificial data clearly showed that *κ* is the most difficult parameter to estimate. This behavior was magnified in the simulations carried out in a systematic way. However, in practice, it is far less prominent, and does not hamper the classification between fast and low release as reported below.

As for the estimation of *D*, results reported in Fig. 3 are very good when *κ* is high enough. Indeed, this behavior is not a surprise, since, for low *κ*, the flow between *C*_*s*_ and *C*_*d*_ is very small. Consequently, few proteins are available to estimate *D* (precisely the ones undergoing a Brownian motion). On the contrary, when increasing *κ*, the estimation becomes more and more accurate as the amount of signal available to estimate *D* increases. When *κ*>0.25, with a MALE of 0.03, estimation of *D* is as precise as the best estimation method for the simpler point source model [17]. Including the worst estimates, the overall MALE for the diffusion coefficient is still very low at 0.06.

### Comparison of TfR and Langerin dynamics

#### Cells and acquired images

**Fig. 4:**
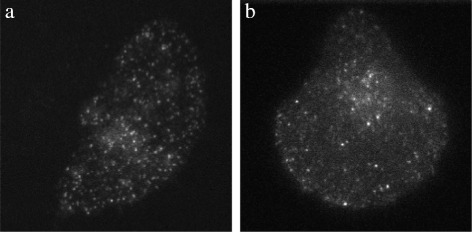
Two sample images from real TIRFM image sequences depicting a micro-patterned cell: **a** TfR proteins, **b** Langerin proteins

We have applied the proposed detection and estimation algorithm to sixteen real TIRFM image sequences of M10 cells, half of which depicting TfR, the other half depicting Langerin. Two sample images are shown in Fig. 4.

**Fig. 5:**
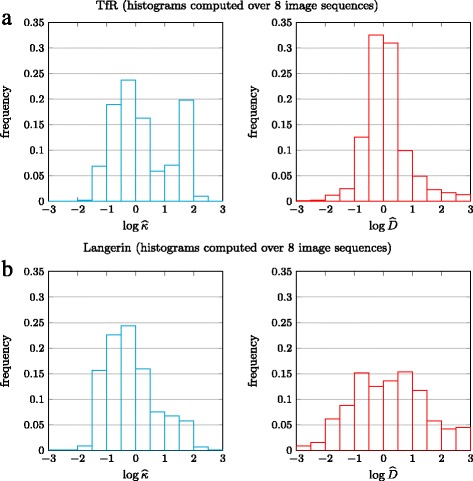
Comparison of the histograms of the biophysical parameters *κ* and *D* estimated respectively in 8 TIRFM image sequences depicting TfR (**a**) and in 8 TIRFM image sequences depicting Langerin (**b**)

The M10 human melanoma cell line and its derivative expressing the Langerin protein have been described previously [35]. Briefly, the CD207 cDNA was cloned in the plasmid pEYFP-C3 (Clontech, Ozyme, Paris, France). The stable M10-Lang-YFP cell line was obtained by transfection of M10 cells using Fugene 6 reagent (Roche Applied Science, Meylan, France) followed by selection of the clones with 400 *μ*g/mL G418 (Invitrogen Fischer Scientific, Illkirch-Graffenstaden, France). Cells are grown in Roswell Park Memorial Institute medium (RPMI) 1640 supplemented with 10% heat-inactivated fetal calf serum (FCS), penicillin and streptomycin (Invitrogen Fischer Scientific). M10 cells were also transiently transfected with plasmid coding for TfR-pHluorin, using the following protocol: 2 *μ*g of DNA, completed to 100 *μ*L with RPMI (FCS free) were incubated for 5 minutes at room temperature. 6 *μ*L of X-tremeGENE 9 DNA Transfection Reagent (Roche Roche Applied Science, Meylan, France) completed to 100 *μ*L with RPMI (FCS free) were added to the mix and incubated for further 15 min at room temperature. The transfection mix was then added to cells grown one day before and incubated further at 37 °C overnight. Cells were then spread onto fibronectin Cytoo chips (Cytoo Cell Architect) for 4 h at 37 °C with RPMI supplemented at 10% (vol/vol) of FCS, 10mM Hepes, 100 units/ml of penicillin and 100 *μ*g/ml of Strep before imaging.

Cells were then imaged using a TIRFM setup based on a Nikon Ti Eclipse equipped with an azimuthal iLas2 TIRFM module (Roper Scientific), a 100x Nikon TIRFM objective (NA 1.47) and an Evolve 512 EMCCD camera. The images were acquired in “stream” mode at 100 ms exposure time per frame. In the set of sequences depicting TfR, 3,147 fusion events are detected, and in those depicting Langerin, 4 223 fusion events.

#### Experimental results

The results are gathered in Fig. 5 in the form of four histograms of $\log \widehat {\kappa }$ and $\log \widehat {D}$, estimated in the sequences depicting TfR or Langerin.

The $\log \widehat {\kappa }$ histograms of TfR and Langerin have very different shapes. While the same first mode is present around 1 f^−1^ for both proteins, the histogram of TfR exhibits another strong peak around 100 f^−1^, comprising as much as 20% of TfR events. This second peak does not appear in the histogram of Langerin. In contrast, much more slow-release events are found in Langerin sequences, around 0.1 f^−1^.

These results are consistent with those reported in [36], in which a simple 1D+time intensity signal was used to classify fusion events as *slow* or *fast*. However, our model and method supply a dramatically improved description of the fusion process, with a complete parameter distribution. Moreover, we supply estimates of biophysical parameters instead of the image-related parameter of [36].

A second conclusion can be drawn, regarding the diffusion coefficient statistics. Indeed, Langerin shows a much higher dispersion of the estimates than TfR. To our knowledge, this was never shown in the frame of vesicle fusion. Indeed, this could not even be analyzed in previous works [16, 36]. In [36], the diffusion was not estimated, while the model used in [16, 17] was too simple to cope with slow-release events. Furthermore, as reported in [17], even for fast-release events, the estimation of *D* was not accurate in [16].

## Conclusion

We have proposed an original dynamical model, called SSED, to represent the vesicle fusion to plasma membrane at the end of the exocysotis process. It includes two biophysical parameters, namely the release rate and diffusion coefficient, and we have developed a method to estimate them in TIRFM image sequences. After demonstrating the efficiency and accuracy of the method on simulated sequences, we successfully applied it to real TIRFM images depicting TfR and Langerin proteins. The experiments demonstrated that the release rate and diffusion coefficient distributions of the two transmembrane proteins clearly exhibit different behaviors to be further explained by biological studies.

The proposed method could still be improved in several directions. Instead of the quadratic criterion (12) used to estimate the SSED model parameters, we could resort to a robust estimation. Indeed, if the background cannot be fully removed, a robust penalty would prevent from biased estimation. Resorting to robust statistics [37], e.g., M-estimators as Hampel, Huber of Tukey’s function, would enable to correclty estimate the model parameters even in presence of outliers, i.e., irrelevant pixels, in the estimation support. If the release rate is very slow, taking cuboids $\mathcal {V}_{i}$’s with a longer temporal dimension would be beneficial. Then, it would be helpful to design an automatic adaptation of the size of the space-time neighborhoods $\mathcal {V}_{i}$’s. Finally, other proteins intervene in the exocytosis process. Among them, Rab11/Rab11-FIP (Rab11 family of interacting proteins) complexes together with cortical cytoskeleton elements, play a crucial role on the internal faces of the carrier vesicles and plasma membrane. However, in order to dynamically relate fusion-diffusion steps of the transported membrane proteins to the release mechanism of these peripheral membrane associated complexes, diffusion studies might be not restricted to 2D, and 3D TIRFM image sequences [38] will then be necessary.

## Abbreviations

EYFP: Enhanced yellow fluorescent protein
FCS: Fetal calf serum
FRAP: Fluorescence recovery after photobleaching
ICS: Image correlation spectroscopy
MALE: Mean absolute logarithmic error
MAP: Maximum a posteriori
MSD: Mean squared displacement
PSF: Point spread function
RPMI: Roswell park memorial institute medium
SPT: Single particle tracking
SSED: Small-extent source with exponential decay release
STICS: Spatio-temporal image correlation spectroscopy
TfR: Transferrin receptor
TIRFM: Total internal reflection fluorescence microscopy
